# Cap Is the Protease of the Porcine Circovirus 2

**DOI:** 10.3390/v14071550

**Published:** 2022-07-15

**Authors:** Xuechen Yang, Wei Yang, Wei Zhang, Jiamei Li, Guoyu Yang, Shuhong Zhao, Yueting Zheng

**Affiliations:** 1 Key Laboratory of Animal Biochemistry and Nutrition, Ministry of Agriculture and Rural Affairs and Key Laboratory of Animal Growth and Development of Henan Province, College of Veterinary Medicine, Henan Agricultural University, Zhengzhou 450046, China; yangtutudexiaohua@hotmail.com (X.Y.); anryest@hotmail.com (W.Z.); lijiamei1106@126.com (J.L.); 2 Key Lab of Agricultural Animal Genetics, Breeding and Reproduction of Ministry of Education and Key Laboratory of Swine Genetics and Breeding of Ministry of Agriculture, College of Animal Science and Technology, Huazhong Agricultural University, Wuhan 430070, China; 02yangwei@163.com

**Keywords:** porcine circovirus type 2, viral capsid, protease, JMJD6, CCT5

## Abstract

Circoviruses are the smallest single-stranded DNA viruses that infect mammalian species, avian species, fish, and insects. The infections of circoviruses are known to be associated with a series of fatal diseases, but the protease of circovirus still remains unknown. In this research, we identified viral capsid protein (Cap) as the protease of porcine circovirus type 2 (PCV2), to our knowledge the first circoviruses protease to be reported. First, we found that the expression of host proteins is affected due to PCV2 infection in the porcine kidney (PK-15) cells. Then, by proteomic analysis, 253 host proteins that were down regulated were identified due to direct or indirect effects of PCV2. Further, Cap expression, but not other ORFs of PCV2, significantly reduced both JMJD6 (bifunctional arginine demethylase and lysyl-hydroxylase) and CCT5 (the chaperonin containing TCP1 subunit 5) in PK-15 cells. Finally, the results in vitro hydrolysis assays demonstrated that Cap could directly degraded either JMJD6 or CCT5 with different catalytic efficiency. In summary, our study expands repertoire of PCV2 Cap and promotes the development of inhibitors toward the anti-PCV2.

## 1. Introduction

Porcine circoviruses (PCVs) in swine have been divided into four different serotypes, Porcine circovirus type 1 (PCV1), Porcine circovirus type 2 (PCV2), Porcine circovirus type 3 (PCV3), and Porcine circovirus type 4 (PCV4) [1]. Among the PCVs, PCV2 is ubiquitous in global scale and is considered to be the major, causative pathogen that causes a series of porcine circovirus-associated diseases (PCVAD) [2,3,4]. Therefore, PCV2 undoubtedly is economically challenging agent that leads to enormous economic loss to the global pig industry.

The PCV2 is small, non-enveloped, icosahedral virus with a single-stranded circular DNA of about 1900 bases. There are eleven potential open reading frames (ORFs), but only five viral proteins were identified so far. ORF1 encodes replicase proteins that are necessary for DNA replication [5]. ORF2 encodes Capsid protein (Cap) that is the sole structural protein and the main immunogenicity of PCV2 [6]. It is reported that Cap is found not only in the cytoplasm and but also the nucleus, indicating that Cap can transport and locate in different cellular compartments during infection. Additionally, Cap can also produce host cell autophagy and induce cell death in porcine kidney cells (PK-15). ORF3, ORF4 and ORF5 are also identified recently. It has been reported that ORF3 induces apoptosis, ORF4 inhibits apoptosis, and ORF5 can stimulate endoplasmic reticulum stress and activate NF-κB signaling pathway [7,8,9]. Although intensive research has been carried out and great progress has made, no protease has been reported among the known coding proteins of PCV2.

Protease, which is ubiquitous in living organisms, plays a fundamental bio-catalytic role in living organisms by directly regulating protein metabolism [10]. Protease genes account for about 6% in the human genome and about 1–5% in pathogenic organisms. The viral proteases are essential for viral replication and particle assembly [11,12,13,14,15,16,17]. In addition, the viral protease was also found mediate viral infectivity by degrading host restriction proteins. Up to now, viral proteases have been found in many varieties of viruses including non-enveloped single-strand RNA viruses (picornaviruses) [11,12], enveloped single-strand RNA viruses (retroviruses, flaviviruses) [13,14], non-enveloped double-strand DNA viruses (adenoviruses) [15], and the enveloped and non-enveloped double-strand DNA viruses (herpesviruses) [16]. However, the protease of PCV2 was still unknown, and it prevents the understanding of PCV2 life cycle processes and the intercellular function and mechanism.

To test which protein is the protease, we expressed all of the five ORFs of PCV2 and examined their proteolysis activity; only Cap is identified. In addition, proteome analyses were carried out and 253 putative host proteins were down regulated due to the effect of the PCV2. The gene ontology annotation was plotted, and most of these host proteins were mainly involved in cell growth and division. Furthermore, arginine demethylase and lysine hydroxylase (JMJD6), which plays an important role in regulating cell processes and fate, and chaperon containing TCP1 subunit 5 (CCT5), which is required for normal cell growth and division, were demonstrated to be down-regulated in vivo assay and be directly degraded by PCV2 Cap in vitro assay. In summary, we provide the first report that Cap is PCV2 protease and directly degrades host proteins JMJD6 and CCT5. Our findings should be helpful in understanding the pathogenic mechanism of PCV2 infection.

## 2. Materials and Methods

### 2.1. Materials

*E. coli* strain TOP10 was purchased from TIANGENBIOTECH Co., Ltd. (Beijing, China) and was used for recombinant plasmid amplification. *E. coli* strain BL21 (DE3) (Vazyme, Nanjing, China) was preserved in the laboratory and was used for protein expression. Porcine Kidney cells (PK-15) was preserved in the laboratory. PK-15 cells were cultured in Dulbecco’s Modified Eagle Medium (DEME) (Corning Inc., NY, USA). All medium were supplemented with 10% (*v*/*v*) fetal bovine serum (FBS) (Gibco, New York, NY, USA). The PCV2 strain (GenBank ID: GU325754), stored in our laboratory. The plasmid pET21b (Novagen, Madison, WI, USA) harboring a C-terminal 6 × His tag was used for protein purification and was preserved in the laboratory. The gene cap, JMJD6 and CCT5 were synthesized by GenScript (Nanjing, China). High-affinity Ni-NTA resin was obtained from GenScript. Lysis buffers were purchased from Beyotime Ins.Bio (Shanghai, China). T4 DNA polymerase, Xho I and BamH I restriction enzymes were purchased from Thermo Fisher Scientific (Shanghai, China); Pfu DNA polymerase was purchased from GenStar (Shanghai, China). The transfection reagent was T101-01/02, Vazyme Biotechnology, Nanjing, China). Other chemicals used were purchased from Sigma-Aldrich (Shanghai, China).

### 2.2. Plasmids Construction

The 624 bp DNA sequence encoding the 208 amino acids of mature PCV2-Cap protein was codon-optimized for *E. coli* expression and synthetized into the pUC57 vector. The target gene was PCR-amplified and then cloned into pET21b expression vector with an N-terminal 6His-EDA tag (pET21b-6His- EDA-PCV2Cap). The expression vector for Cap was designed for metal (Ni^2+^) affinity purification with 6His. The recombinant plasmids (pET21b-6His-JMJD6 and pET21b-6His-CCT5) were also constructed in the same way, respectively. The constructed plasmids were confirmed by DNA-sequencing in Sangon Biotech (Shanghai, China) Co., Ltd.

To construct pcDNA-flag-Cap, the gene of PCV2 Cap were amplified from the genomic DNA of the PCV2 with the synthetic primers. Then the PCR product of Cap was digested using the EcoRI and BamHI restriction enzymes and cloned into the multiple cloning site of pcDNA3.1. Other ORFs of PCV2 were constructed in the same way. The constructed plasmids were confirmed by DNA-sequencing in Sangon Biotech (Shanghai, China) Co., Ltd.

### 2.3. The Analysis of Host Protein Proteome

PK-15 cells, mock infected or infected with PCV2, were incubated at 37 °C for 12 h. Then the PK-15 cells were harvested and lysed in lysis buffer (100 mM Tris HCl, pH 8.0, 100 mM NaCl, 1% sodium deoxycholate, 1% Triton X-100). After homogenization, the whole-cell lysates were centrifuged for 10,000 rpm, 30 min at 4 °C and centrifuged for 25,000× *g*, 30 min at 4 °C to remove the cellular debris. The samples were used in proteomic detection in Shenzhen BGI Co., LTD (Guangzhou, China).

### 2.4. Proteomics Analysis

To identify signaling pathway involved in the down-regulated proteins, the KEGG analysis (https://www.genome.jp/kegg/pathway.html, accessed on 23 May 2022) was mapped (*p* value < 0.05). Then the down-regulated proteins were searched against Gene Ontology database (http://geneontology.org/, accessed on 23 May 2022) to obtain enrichment information of molecular function, cellular component, and biological process [18]. To further illuminate the relationship between these down-regulated proteins, protein-protein interactions (PPI) analysis was applied and showed (PPI enrichment *p* value < 0.05) [19].

### 2.5. Cell Transfection

For transfection, PK-15 cells were seeded in monolayers onto the plates and maintained in DMEM with 10% heat-inactivated FBS at a suitable density. When the PK-15 cells grow to about 80% confluences, the confirmed plasmids were transfected into PK-15 cells using Lipofectamine 3000 (Invitrogen, Grand Island, NY, USA).

### 2.6. SDS-PAGE and Western Blotting

For Western blotting, the samples were separated by standard SDS-PAGE and then transferred to polypropylene fluoride membranes (GE Healthcare, Beijing, China). Membranes were followed by blocking with PBS containing 5% nonfat milk for 1 h and then washed with PBS. After incubated with primary antibody, membranes were incubated with the mouse monoclonal anti-JMJD6 (1:1000), mouse monoclonal antibodies to CCT5 (1:1000), and anti-β-actin (1:1000) overnight at 4 °C. All antibodies used were tested with well specificity. After incubation with anti-mouse horseradish peroxidase-labeled antibody for another hour, blots were revealed by the GE AI600imaging system (GE Healthcare).

### 2.7. Protein Expression and Purification

A single colony was picked from an agar plate and inoculated in 4 mL liguid Luria-Bertain medium (LB) containing ampicillin (100 μg/mL, Amp) overnight at 37 °C with constant shaking at 220 rpm. Protein expression was induced with adding 0.5 mM/L isopropyl-beta-D-thiogalactopyranoside (IPTG) and the cells were further cultured for 12 h at 18 °C, 220 rpm. Then the cells were harvested by centrifugation (3000× *g*) at 4 °C for 30 min. The pellets obtained were re-suspended in 10 mL lysis buffer (50 mmol·L^−1^ Tris-HCl, 30 mmol·L^−1^ NaCl, 10 mmol·L^−1^ imidazole, pH 7.5). The re-suspended cells were sonicated and then centrifugated at 12,000× *g* for 30 min to remove cell debris and insoluble contaminants. The EDA-Cap supernatants were loaded to the Ni-NTA column for the purification of the target protein. The Ni-NTA columns were washed with Wash Buffer (50 mmol·L^−1^ Tris-HCl, 30 mmol·L^−1^ NaCl, 80 mmol·L^−1^ imidazole, pH 7.5) to remove non-specific binding proteins, and the target protein with His-tag was eluted using Elution Buffer (50 mmol·L^−1^ Tris-HCl, 30 mmol·L^−1^ NaCl, 300 mmol·L^−1^ imidazole, pH 7.5). The collected samples were detected by 12% polyacrylamide gel electrophoresisfor (SDS-PAGE). JMJD6 and CCT5 were also expressed and purified as the same way, respectively.

### 2.8. Enzymatic Activity Assay of the Cap with the Host Proteins

The enzymatic activity of PCV2 Cap on the host proteins (JMJD6 and CCT5), which were identified by proteome, was further verified. The host proteins (JMJD6 and CCT5) were expressed and purified as described above. The reaction mixture (50 µL) contained 50 mmol·L^−1^ Tris-HCl, 5 mmol·L^−1^ KCl, 5 mmol·L^−1^ (NH_4_)_2_SO_4_, 2 mmol·L^−1^ MgSO_4_, 20 uM substrate, and 3 uM Cap. Reactions were performed in the Eppendorf tubes and incubated at 37 °C for 30 min and then analyzed by SDS-PAGE.

### 2.9. Statistical Analysis

The results obtained from at least three independent experiments were presented as means ± the standard deviations and statistical analyzed with the *t* tests. Significant analyses of differences were accepted at *p* values of < 0.05.

## 3. Results and Discussion

### 3.1. Proteomic Analysis of Host Proteins in PK-15 Cells Infected by PCV2

**Figure 1:**
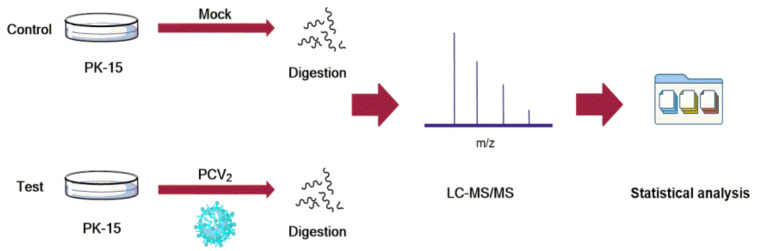
Experimental design.

**Figure 2:**
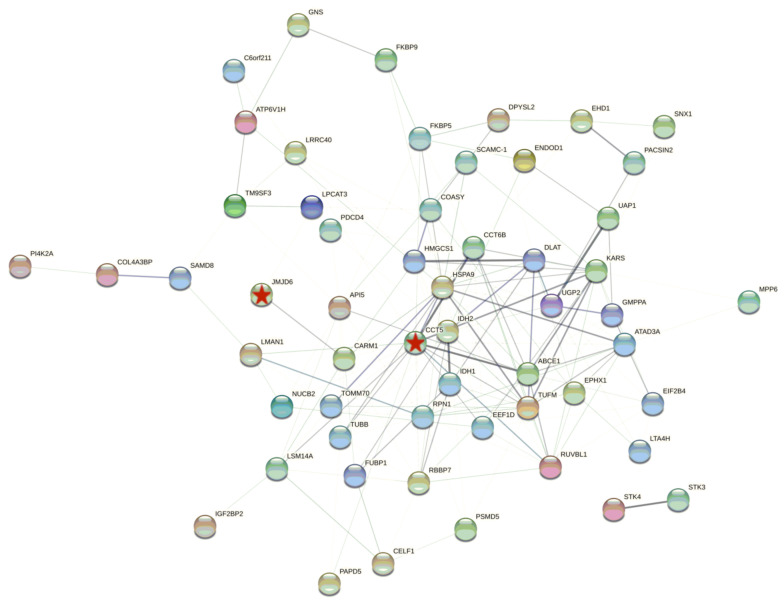
The protein-protein interactions (PPIs) network analysis using STRING database. The linkers indicate associations of function and physical protein. The thickness of line indicates the strength of correlation (PPI enrichment *p* value < 0.05). Proteins represented have been named as NCBI gene names. JMJD6 and CCT5 were marked with Stars.

**Figure 3:**
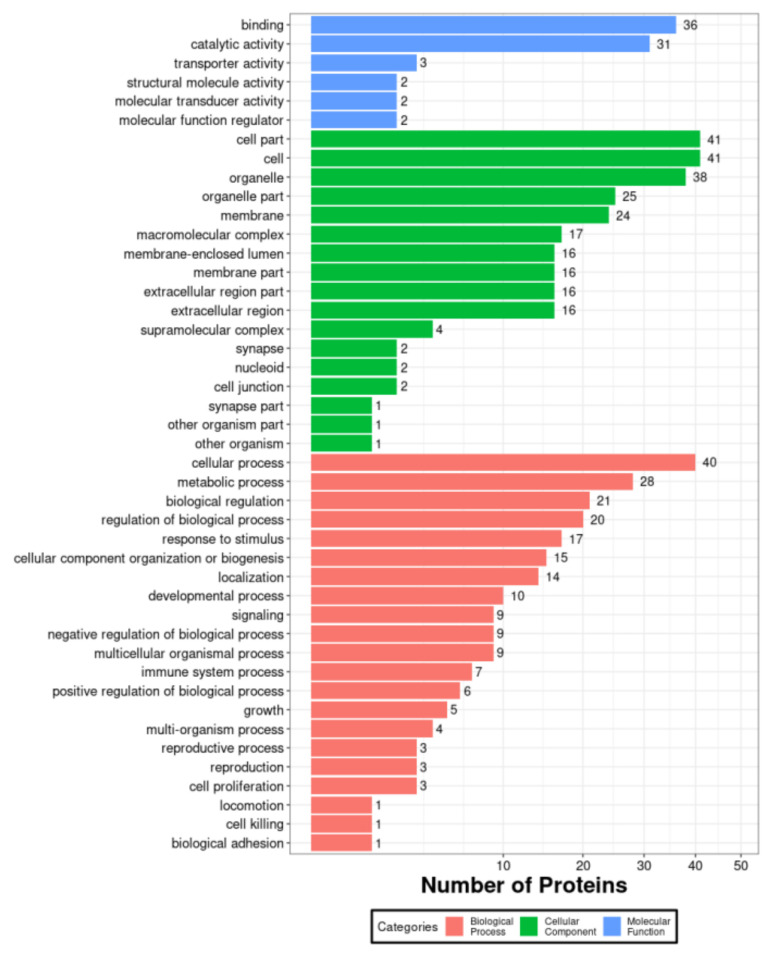
GO enrichment analysis of the top 66 down-regulated proteins. The distribution is divided into three categories: biological process, molecular function, and cellular component in 66 down regulated proteins. The *x*-axis indicates the number of proteins and the *y*-axis indicates different GO terms.

PK-15 cells have been commonly used in the research of the pathogenic mechanism of PCV2 infection. To investigate the host proteins affected by PCV2, we performed proteomic analysis of PK-15 cells. The PK-15 cells, mock infected or infected by PCV2, were prepared and then were subjected to proteomic assays (Figure 1). The results showed that about 1942 and 1963 proteins were identified in mock infected and infected group, respectively. The Venn diagram showed the identified host proteins of PK-15 cells in response to the infection of PCV2. There were 253 host proteins that were down-regulated and there were 122 host proteins that were up-regulated due to PCV2 infection. Among them, the top 10 down-regulated and up-regulated host proteins in PK15 cells in response to PCV2 were shown in Table 1 and Table 2, respectively. To understand the relationship between the down-regulated host proteins, protein-protein interactions (PPI) network was constructed using STRING database and represented in Figure 2. To further illuminate and predict the host cell proteins those have been degraded by the PCV2 Cap, we performed gene ontology (GO) annotation and predicted its biological functions. GO annotation and analysis of the top 66 down-regulated proteins were represented to three categories: molecular functions, cellular components, and biological processes (Figure 3). The GO terms of molecular function category were concentrated in “binding” (36 out of 66) and “catalytic activity” (31 out of 66). The GO terms under cellular component class were “cell part” (41 out of 66) and “cell” (41 out of 66). Besides, the GO terms under biological process were “cellular process” (40 out of 66) and “metabolic process” (28 out of 66). Collectively, these findings inferred that the PCV2 infection would significantly perturb several cellular processes such as metabolism, protein interactions, cell processes and fate, as well as cell growth and division.

### 3.2. Validation That Down-Regulation of Host Proteins Is Due to Cap

**Figure 4:**
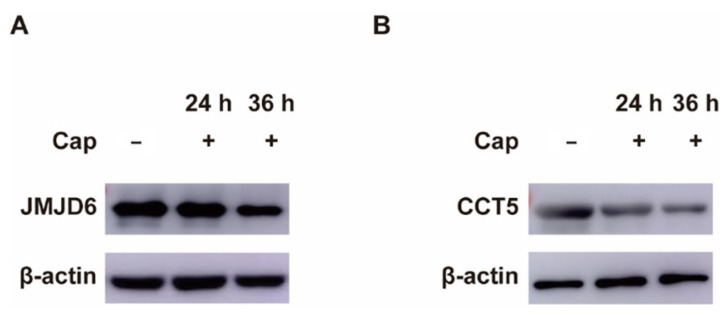
We validated the PCV2 Cap down-regulated host proteins by Western blotting (WB). (**A**) The expression of JMJD6 in PK-15 cells was determined by WB analysis. (**B**) The expression of CCT5 in PK-15 cells was determined by WB analysis.

To validate the specific PCV2 protein involved in the down-regulation of host proteins, we expressed each ORF of PCV2 in PK-15 cells and investigated its effects on host proteins, JMJD6 and CCT5. Both JMJD6 and CCT5 are essential for normal cell processes and decreased in proteomic assay. As shown in Figure 4, Cap caused significant reduction of JMJD6 and it was in time-dependent manner (Figure 4A). CCT5 was also reduced when Cap was expressed (Figure 4B). Moreover, other ORFs of PCV2 could not cause the decrease of JMJD6 and CCT5. These results suggested that Cap is primarily responsible for down-regulating host proteins during PCV2 infection.

### 3.3. Validation That Cap down Regulates Host Proteins by Directly Hydrolyzing

**Figure 5:**
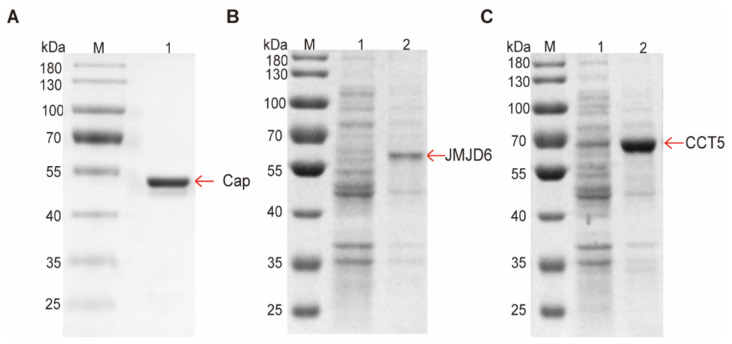
Expression and purification of Cap, JMJD6 and CCT5. (**A**) SDS-PAGE analysis of the purified EDA-Cap. M, protein molecular mass standards; 1, the purified EDA-PCV2 Cap. (**B**) SDS-PAGE analysis of the purified JMJD6. M, protein molecular mass standards; 1, soluble fraction after cell sonication; 2, the purified JMJD6. (**C**) SDS-PAGE analysis of the purified CCT5. M, protein molecular mass standards; 1, soluble fraction after cell sonication; 2, the purified CCT5.

**Figure 6:**
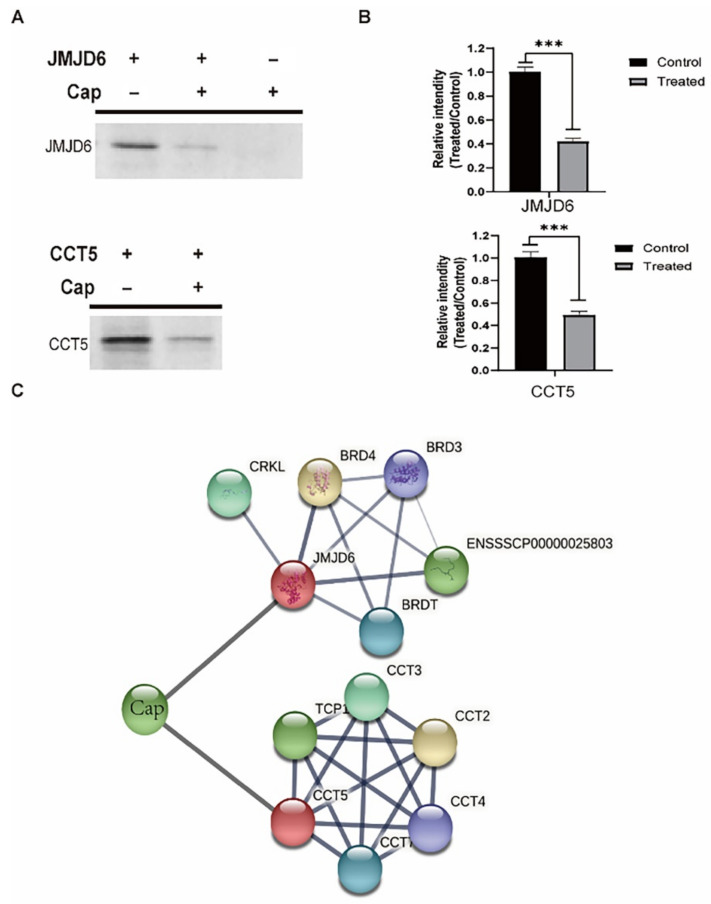
The in vitro reaction assays and the PPI networks. (**A**) SDS-PAGE analysis the in vitro reaction that Cap digested JMJD6 and CCT5 at 37 °C 30 min, respectively. (**B**) Statistical analysis of the deduction of JMJD6 and CCT5 due to the digestion of Cap. All data were obtained from at least three independent experiments (*p* < 0.05, *p* < 0.01, *** *p* < 0.001). (**C**) The PPI networks of the PCV2 Cap with JMJD6 and CCT5.

In order to identify that the decrease of JMJD6 and CCT5 is the direct effect of Cap, we conducted in vitro reaction assays. First, two host proteins, JMJD6 and CCT5, and Cap were expressed and purified in *E. coli*, respectively (Figure 5). Then the group without Cap was prepared as a negative control and the group with Cap as test group. SDS-PAGE results showed that the bands of either JMJD6 or CCT5 were significantly decreased when Cap was added. Quantification of the enzymatic activity of recombinant EDA-Cap was analysed by Image J and GraphPad Prism version 8.00 (GraphPad Software, San Diego, CA, USA) (Figure 6). As shown in Figure 6B, JMJD6 decreased by 58.69% and CCT5 decreased by 50.09% and these results were consistent with the proteome results. These results indicated that the hydrolysis efficiency of Cap to JMJD6 was higher than that of CCT5. Taken together, these results confirmed that PCV2 Cap could directly digest host proteins and the catalytic efficiency of Cap varies with different host proteins. Then, for further understanding the biological relationships of the confirmed host proteins hydrolyzed by Cap, JMJD6 and CCT5, we constructed another PPI network and it showed five extra interacting proteins, TCP1, CCT2, CCT3, CCT4 and CCT7. (Figure 6C). Consistent with these results, we concluded that by reducing host proteins, PCV2 infection disrupted normal cell processes and Cap hydrolyzed host proteins directly.

## 4. Conclusions

Porcine circovirus type 2 (PCV2) is the main pathogen causing porcine circovirus-associated diseases and leads to continuous infection and immunosuppression in pigs. It causes huge economic losses to the global pig industry and brings with it a substantial economic burden. Since PCV2 was discovered in 1998, great progress has been made. However, the molecular mechanism of PCV2 interacting with the host proteins and its pathogenesis are still rarely understood and PCV2 protease hasn’t been identified yet. Actually, the virus proteases are ubiquitous and play the crucial roles in the virus infection. The alphavirus contains an icosahedral nucleocapsid and the capsid of Alphavirus has been identified to be a virus protease. It is hypothesized that PCV2 capsid may be the protease and perhaps affect various stages of cell cycle by proteolysis.

In this study, the host proteins of PK-15 cells affected by PCV2 were analyzed by proteomic assay for the first time. We have screened 253 putative cellular proteins which could be substrate of Cap according to the qualitative analysis proteomics. These host proteins play essential roles in cell processes, metabolic regulation, and immune response. Therefore, the host proteins identified in this research provide new ways for elucidating the pathogenesis of PCV2. Among them, we have also identified JMJD6 and CCT5, which could be down-regulated in vivo and hydrolyzed directly in vitro by Cap. JMJD6 is one of the members of the Jumonji C (JmjC) domain-containing protein, a class of demethylase enzymes. Its biological function is mainly to regulate gene expression, including the roles in cell growth and differentiation, cell proliferation, and stress response. In this study, we found the significant reduction of JMJD6, which catalyzes lysine de-methylation of histones. These results suggest that PCV2 may affect histone methylation modifications through the enzymatic hydrolysis of JMJD6 by Cap, thereby affecting cell growth and reproduction. This study also showed the reduction of the CCT5 which is one of the subunits of the chaperon containing TCP-1 (CCT, or TRiC). It is reported that CCT regulates cell cycle progression and cytoskeletal organization due to its essential role in the folding of many important proteins. Therefore, the results of CCT5 reducing indicated that PCV2 probably damaged the cell division by proteolysis CCT5 by Cap. PK-15 cells infected porcine circovirus contain many intracytoplasmic inclusion bodies. A few infected cells contained intranuclear inclusion bodies. Cap-induced unfolded protein response (UPR) leads to apoptotic responses via the PERK/eIF2α/ATF4/CHOP/Bcl-2 pathway. Determination of Cap enzyme activity of PCV2 and its interaction with host proteins will contribute to a better understanding of PCV2 pathogenesis. Furthermore, identification of host proteins that are hydrolyzed by PCV2 Cap will help provide new insights into the study of new targets for host effectors or viral factors, and ultimately help design better therapeutic strategies for PCVAD.

## Figures and Tables

**Table 1:** Top 10 downregulated proteins down-regulated by PCV_2_ Cap digestion.

Accession No.	Protein	Functions
tr|A0A287BND4	TOMM70	Innate immune pathway
tr|A0A480ZTV3	ENDOD1	endonuclease activity
tr|A0A4X1W398	LOC100525876	mitochondrion organization
tr|A0A480SUM8	SARS2	serine-tRNA ligase activity
tr|A0A480PJ32	EIF2B4	translation initiation factor activity
tr|F1SHH7	API5	fibroblast growth factor binding
tr|A0A5G2R9Q7	LSM14A	DNA/RNA binding
tr|A0A4X1TKG0	LMAN1	endoplasmic reticulum organization
tr|Q95N04	DLAT	pyruvate metabolic process
tr|A0A287B466	PSEN2	intracellular signal transduction

**Table 2:** Top 10 up-regulated proteins induced by PCV_2_ Cap digestion.

Accession No.	Protein	Functions
tr|A5A758	KRT1	associated with bullous congenital ichthyosiform erythroderma
tr|A0A4X1TWN6	TMX3	protein-disulfide reductase activity
tr|A0A4X1SP89	N/A	actin binding
tr|A0A5G2R9A9	PFN1	actin binding
tr|I3LD72	EHD2	ATP binding
tr|A0A4X1WBN6	N/A	N/A
tr|A0A287AAG0	UAP1L1	UDP-N-acetylglucosamine diphosphorylase activity
tr|A0A4X1V6Q9	DDX39A	ATP binding
tr|A0A480MAF3	MRPL16	rRNA binding
tr|A0A480Z0T5	KLC1	regulate organelle transport
